# Attitude Toward Family Involvement in End-of-Life Care Among Older Chinese Americans

**DOI:** 10.1093/geroni/igaa057.1638

**Published:** 2020-12-16

**Authors:** Kaipeng Wang, Yanqin Liu, Fei Sun, Dexia Kong, Bei Wu

**Affiliations:** 1 University of Denver, Denver, Colorado, United States; 2 Arizona State University, Tempe, Arizona, United States; 3 Michigan State University, East Lansing, Michigan, United States; 4 Rutgers University, New Brunswick, New Jersey, United States; 5 New York University, New York, New York, United States

## Abstract

Family involvement is critical to end-of-life (EOL) care of older adults. Attitude toward family involvement in EOL care can be influenced by family relationship. Yet, mechanisms explaining such influence have not been examined among older Chinese Americans. This study aims to examine the association between family relationship and older Chinese Americans’ attitude toward family involvement in EOL care and explore pathways of this association. Potential mediators include self-efficacy, perceived benefits, and perceived barriers of discussing EOL care with family members. Data were collected from 276 Chinese Americans aged 55+ in two metropolitan areas in 2018. Participants’ average age was 74 years (SD=9.6). Approximately 64% of the sample were female. Most participants (57%) held positive attitudes toward family involvement in EOL care. Using the Structural Equation Modeling method, we found that family relationship had a significant positive total effect on positive attitude toward family involvement in EOL care (z=5.57, p<0.001). Indirect direct of family relationship on attitude toward family involvement in EOL care through both self-efficacy (z=3.13, p<0.01) and perceived barriers (z=2.30, p<0.05) of discussing EOL care with family members was significant. Results suggest that improving family relationship may increase elder’s self-efficacy and reduce barriers of discussing EOL with family members, which is associated with more positive attitude toward family involvement in EOL care. Findings provide empirical evidence of how family relationship affects older Chinese Americans’ attitude toward family involvement in EOL care and underline the need for family-centered interventions for older Chinese Americans.

